# Potential Utility of Cell Free High Mobility Group AT-hook 2 (*HMGA2*) as a Prognostic Biomarker in Liquid Biopsies of Oral Squamous Cell Carcinoma

**DOI:** 10.31557/APJCP.2021.22.2.407

**Published:** 2021-02

**Authors:** Nosheen Mahmood, Shamim Mushtaq, Qamar Jamal, Muhammad Hanif, Humera Akhlaq, Dur-e-Shewar Rehman, Rashid Awan

**Affiliations:** 1 *Ziauddin University, Pakistan. *; 2 *Biochemsitry, Director Postgraduate Ziauddin University, Pakistan. *; 3 *Karachi Institute of Radiotherapy and Nuclear Medicine, Pakistan. *; 4 *Jinnah Sind Medical University, Pakistan. *; 5 *Anatomy, King Saud Bin Abdul Aziz University, Saudi Arabia.*; 6 *Internal, Medicine, Chinniot General Hospital, Pakistan. *

**Keywords:** OSCC, oral cancer, HMGA2, liquid biopsy, plasma

## Abstract

**Background:**

Locoregional spread is a frequent finding in oral cancer which dictates poor prognosis. HMGA2 expression has been linked to malignant traits of oral cancer in tissue biopsies however, data on HMGA2 expression in liquid biopsies in oral cancer is sparse. Purpose of this study was to explore prognostic relevance of HMGA2 in liquid biopsies of oral cancer patients.

**Patients and Methods:**

After obtaining approval from Institutional Review Board of Ziauddin University and informed written consent from study subjects, expression of circulating HMGA2 was evaluated in 96 OSCC cases and 100 age and sex matched controls via real time PCR using specific set of primers. We further analyzed relationship of various sociodemographic and clinicopathological variables with HMGA2expression and explored its prognostic potential.

**Results:**

Expression was seen in 22 (23%) cases. A higher expression was observed among subjects with local invasion (52.6% vs 47.4 %), distant metastasis (71.4% vs 28.6%) and tumor recurrence (57.1% vs 42.9%) p <0.05. Subjects having HMGA2 expression had a poor survival compared to HMGA2 negative (13.6% vs 35.4%), p <0.05.

**Conclusion:**

Circulating HMGA2 reflects presence of local invasion and distant metastasis and dictates poor prognosis in OSCC. It may contribute in categorizing high risk patients using a minimally invasive technique who are likely to benefit from targeted therapy.

## Introduction

HMGA2, a Let 7 microRNA target, is a member of protein family, termed High Mobility Group (HMG). It regulates expression of genes involved in cell growth, apoptosis and cancer formation. This unique ability is attributed by presence of three short basic repeats called AT- hooks which allow them to pair with AT-rich DNA sequences of many transcription factors (Galdiero et al., 2015). Most attention-grabbing feature of *HMGA2* is its expression only in the phase of active cell division be it physiological (Embryogenesis) or pathological (carcinogenesis) and hence no expression is expected in normal adults. This time dependent regulation is mediated by Let- 7 miRNA which keeps a check at HMGA2. Let 7 (Lethal-7) a tumor suppressor miRNA is downregulated in many malignancies with a resultant unchecked expression of *HMGA2* and hence tumor progression (Mayr et al., 2007). 

There is a huge discrepancy in geographical distribution of oral cancer. While it does not stand in the list of top 10 malignancies worldwide the scenario is very dissimilar in Pakistan where it is the most common cancer amongst men and second only to breast amongst women. It remains a significant health concern in Pakistan as we are witnessing a rising incidence, mortality (IARC Globocan., 2018), and poor prognosis with majority of patients presenting at an advanced stage (Seo et al., 2016). This mandates discovery of tools capable of predicting prognosis by utilizing minimally invasive techniques. 

Overexpression of this oncofetal transcription factor (HMGA2) has been reported in various human malignancies including head and neck, breast, lung, thyroid, ovary and pancreas (Galdiero et al., 2015). It instructs poor overall survival and disease-free survival in head and neck cancers (Huang et al.,2018). It also effects chemo sensitivity in tumors via its interaction with the human sub telomerase activity (Ahmed et al., 2019). It is therefore predicted to categorize cancers in terms of prognosis and treatment response thus opening up new avenues for exploiting possible therapeutic targets. 

While its expression in tissue biopsies from head and neck cancers has been confirmed but its capability of predicting prognosis through liquid biopsies remains to be determined. Accumulating evidence on quantifiable detection in plasma adds further scope to its potential of being used as a minimally invasive biomarker (Galdiero et al., 2015). Tumor cells undergo apoptosis and necrosis and shed their nucleic acids in to circulation which may be detected in plasma. This technique becomes extremely valuable for detecting metastasis and recurrence when the primary tumor has already been surgically excised (Dominguez et al., 2018). Furthermore, tumor evolution allows accumulation of sub clones with additional attributes which have a higher probability of being detected in circulation compared to tissue biopsies.

Considering this we aimed to detect circulating HMGA2 in liquid biopsies of oral cancer patients and to dicover its diagnostic and prognostic potential.

## Materials and Methodos

This case control study was carried out after approval by Ethical Review committee and Board of Advanced studies Ziauddin university. Cases were recruited from oncology department of Ziauddin hospital via purposive sampling. Cases were included if they had biopsy proven squamous cell carcinoma and gave informed written consent. They were excluded if there was a history of any other malignancy, stroke, congestive cardiac failure or myocardial infarction. Controls were either healthy family members of cases or randomly selected age and sex matched healthy indivisuals who met inclusion and exclusion criteria. Controls were included if they were non smokers, not using any smokeless tobacco products and gave informed written consent.However, they were exclused if there was any history of malignancy, stroke, congestive cardiac failure or myocardial infarction .Sample size was calculated using OpenEpi software. At confidence level of 99 % and power of 95 % the minimum sample size required was 28 for each group. We did analysis on 96 cases and 100 controls. 

A detailed interview, physical examination and review of records was carried out to collect data on various sociodemographic and clinicpathological variables. Information on smoking, alcohol consumption and various chewable tobacco products was carefully recorded. Type of product, years since addiction and amount of product used was also noted. 

RNA was extracted through Favorgen total RNA extraction kit (catalog No: FAVNR001-2, Lot No: BE 219214318) according to manufacturer’s protocol. cDNA was immediately synthesized using Revert Aid first strand cDNA KIT from ThermoFischer. To ensure proper RNA extraction and effective cDNA synthesis all prepared cDNA were amplified for GAPDH. Only samples showing 496 bp bands of GAPDH were taken for HMGA2 analysis 

*HMGA2 using Real Time PCR protocol*

*HMGA2* expression was checked through real time PCR. Following reagents were added to PCR tubes, SYBR Green master mix 6.75 µl, HMGA2 forward primer1 µl, HMGA2 reverse primer1 µl, Nuclease free H2O2 µl, cDNA sample 2.5 µl to obtain a final a reaction volume of 13 µl. Following set of primers was used for Real time PCR adapted from research by Zhao et al. Forward primer 5’-AAAGCAGCTCAAAAGAAAGCA-3’; Reverse primer 5’-TGTTGTGGCCATTTCCTAGGT-3’. PCR tubes were vortexed and reaction plate was set up. Rox was used to control for back ground noise on PCR program. Thermal profile was set as follows, an initial denaturation at 95ºC for 5 min followed by 45 cycles of denaturation at 95ºC for 20s annealing at 60ºC for 15 s and extension at 72ºC for 20 s. At the end of reaction Ct values for each sample were noted and any expression seen after 35 was considered negative.

*Statistical Analysis:*

All statistical analysis was performed using the SPSS software package SPSS (Version 24.0; SPSS Inc. Chicago, IL, USA). Chi- square test or Fisher’s exact was used for comparison of nominal and ordinal variables between the groups. Symmetric continuous variables were described as mean with standard deviation and examined using Students t test. Binary Logistic regression method was applied to confirm associations of the dichotomized markers with outcome variables. Models were created by including variables having a significance < 0.1 and ≥ 0.05.

Survival estimates were calculated for the time period 2013–2017.Survival curves were generated through Kaplan-Meier and Log rank test was run to compare different factors affecting survival. Statistical significance was set at P<0.05. 

## Results

*HMGA2* expression was checked on prepared cDNA using specific set of primers. Quality of extraction was confirmed on spectrophotometry and appropriate cDNA synthesis was confirmed by checking expression of *GAPDH *taken as internal control. 

Among cases expression was observed in twenty-two cases out of 96 and as per our expectations HMGA2 did not express in any of normal healthy controls. The proportions of subjects in HMGA2 positive vs. negative in regards to age, gender, smoking status, tumor sites, tumor size, nodal involvement and tumor grades did not differ significantly in the two groups (Tables 1 and 2). Comparative analysis of baseline demographics and tumor characteristics of the HMGA2-negative vs. HMGA2-positive groups revealed that the latter group included higher proportion of subjects with local invasion (52.6% vs 47.4 %), distant metastasis (71.4% vs 28.6 %) and tumor recurrence (57.1 % vs 42.9%) p<0.05. Survival rate among HMGA2 patients was 13.6% compared to 35.4 % among HMGA2 negative subjects. 

To confirm these associations binary logistic regression analysis was carried out. A model was created which included local invasion, distant metastasis, recurrence and outcome as explanatory variables. Moreover, any variable with a p value <0.1 and more than 0.05 was also included in this model hence family history of OSCC was included. Absence of local invasion, being alive or censored, absence of distant metastasis and negative family history of OSCC were used as reference category. Of the examined factors, a positive and significant association was confirmed with local invasion (= 0.005), distant metastasis (p=0.006) and outcome(p=0.008). However, no association could be established with positive family history and local recurrence in this model Table 3.

Cigarette smoking was reported by 61(63 %) of patients and 81(84 %) patients of OSCC were addicted to some form of chewing formulations. The relationship between *HMGA2* expression and smoking and chewing habits was analyzed using chi square and Fischer’s Exact test where appropriate as shown in Table 4. It may be concluded that *HMGA2* expression was not correlated with smoking status as well as frequency and duration of smoking. Similarly, it did not impact frequency or duration of chewing smokeless tobacco products. However, it may be observed that 50 % of gutka chewers were HMGA2 positive, compared to this none of pan or Naswar chewers was HMGA2 positive.

Out of 74 patients who did not have *HMGA2 *expression in plasma initially, 9 patients presented with metastatic disease during follow up. A serum HMGA2 was repeated and it expressed in 7 of 9 patients at this stage further confirming a role of HMGA2 in predicting metastatic disease.

*Association of HMGA2 expression with survival*

**Figure 1:**
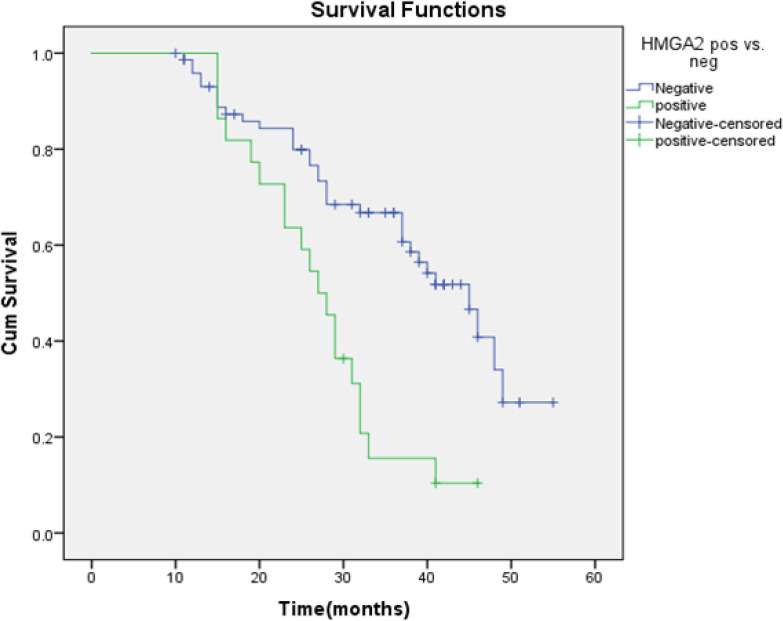
Kaplan Meier Survival Curves of OSCC Patients Having HMGA2 Expression (n=22) vs. no HMGA2 Expression (n=74)

Survival of HMGA2 positive subjects was 13.6% vs. 35.4 % for HMGA2 negative. Kaplan Meier survival function was used to conduct survival analysis and Log rank test was run to compare survival of HMGA vs HMGA2 -ve subjects. Survival was poor for subjects having* HMGA2* expression with a mean survival of 27.64 ±1.98 months (95 % CI: 23.75-31.54) as compared to 39.3± 1.9 months (95 % CI:35.5-43), x^2^ 12.79, p<0.001. Survival plot is seen in Figure 1. 

**Table 1:** *HMGA2 *Expression in Relation to Patient Characteristics

Characteristic	*HMGA2* Status		P value
	Negative 74 (77%)	Positive 22 (22.9%)	
Age (years)	42.50±12.4	45.8± 12.6	0.226
Sex			
Male	50 (75.8%)	16 (24.2%)	0.64
Females	24 (80 %)	6 (20 %)	
Ethnicity			
Urdu speaking	50 (79.4%)	13 (20.6%)	0.46
others	24 (72.7%)	9 (27.3 %)	
Family History			
Yes	4 (50%)	4 (50%)	0.07!
No	70 (79.5%)	18 (20.5%)	

**Table 2:** *HMGA2* Expression in Relation to Ttumor Characteristics

Characteristic	HMGA2 Status		P value
	Negative	Positive	
Tumor site			
Tongue	8 (66.7%)	4 (33.3%)	0.684
Lip	2 (100 %)	0 (0 %)	
Buccal cavity	64 (78.8%)	18 (22%)	
Tumor size			
T1 & T2	37 (77%)	11 (22.9 %)	1
T3 & T4	37 (77%)	11 (22.9 %)	
Nodal involvement			
Negative	7 (100%)	0 (0%)	0.346!
Positive	67 (75.3%)	22 (24.7%)	
Metastasis			
No	72 (80 %)	17 (19.1%)	<0.006*
Yes	2 (28.6%)	5 (71.4%)	
Recurrence			
No	71 (79.8%)	18 (20.2%)	0.046*
Yes	3 (42.9%)	4 (57.1%)	
Loco regional metastasis			
No	65 (84.4%)	12 (15.6%)	0.001**
Yes	9 (47.4%)	10 (52.6%)	
Grade			
I	9 (69.2%)	4 (30.8%)	0.75
II	52 (77.6%)	15 (22.4%)	
III	13 (81.3%)	3 (18.8%)	

ICD 10-00, Lip; ICD 10-02, Dorsal surface of tongue; ICD 10-05, Palate; ICD10-06, Cheek; retromolar and vestibule, *, P<0.05**; P<0.005.

**Table 3:** Binary Logistic Regression Analysis of Variables Associated with *HMGA2*

Variable	OR	95 % CI	P value
Family History of OSCC			
Yes, vs No	0.445	0.055-3.6	0.449
Metastasis			
Yes, vs No	0.063	0.009 - 0.459	0.006
Local invasion			
Yes, vs No	0.158	0.044 - 0.572	0.005
Recurrence			
Yes, vs No	4.83	0.834 - 28.04	0.079
Outcome			
Died vs alive or censored	0.122	0.026 - 0.572	0.008

CI, Confidence Interval; OR, Odd ratio

**Table 4:** Association of *HMGA2* with Smoking and Chewing Habits

	HMGA2 +VE	HMGA2 -VE	P value
Non-Smoker	7 (20%)	28 (80%)	0.62
Smoker	15 (24.6%)	46 (75.4%)	
# of cigarettes/day			
<10	2 (25%)	6 (75%)	0.766!
10-20	7 (20%)	27 (79%)	
>20	6 (31%)	13 (68%)	
Years since smoking			
< 10	1 (9%)	10 (90.9%)	0.093!
10-20	12 (36%)	21 (63%)	
Chewing habits			
Pan	0 (0%)	9 (%)	0.032*!
Gutka	7 (%)	7 (%)	
Naswar	0 (%)	5 (%)	
Chalia	4 (%)	11 (%)	
Multiple formulations	10 (%)	28 (%)	
Non chewers	1 (%)	14 (%)	
Chewing frequency per day			
≤10	11 (%)	46 (%)	0.132!
10-15	6 (%)	12 (%)	
>16	3 (%)	3 (%)	
Years since Chewing			
< 5	10 (%)	18 (%)	0.209
5-10	6 (%)	21 (%)	
>10	4 (%)	22 (%)	

!, Fischers Exact test used; *, P<0.05

## Discussion

To the best of our knowledge this is first attempt to evaluate potential of HMGA2 of being used as a minimally-invasive biomarker in oral Squamous cell carcinoma patients in Pakistan. Only one study has reported its presence in circulation of oral cancer (Ren et al., 2017). HMGA2, a Let7 microRNA target gene is oncofetal, expressed in phase of active cell division and linked to malignant phenotype of different tumors. How it expresses at the right time is dependent on Let 7 miRNA which keeps a check at this oncofetal gene (Lee and Dutta, 2007 ). This makes its expression in an adult a useful tool for detecting cancers. Expression of *HMGA2* has been investigated in many cancers including OSCC in tissue biopsies however its expression in liquid biopsy needs to be explored.

We chose to investigate expression of circulating *HMGA2* as there is little evidence on potential of circulating HMGA2. Only few researches have addressed this approach in ovarian and oral cancer (Ren et al., 2017; Galdiero et al., 2015). Our results indicated that it did express in subjects having local invasion and distant metastasis. We could not find any research conducted on *HMGA2* expression in tissue or liquid biopsy in OSCC from Pakistan. An association of *HMGA2* expression has been reported with locoregional and distant metastasis in tongue cancer (Zhao et al., 2016).

 The proposed mechanism for *HMGA2 *expression observed in tumor having local invasion is epithelial mesenchymal transition (EMT). EMT is a critical process involved in embryogenesis and tumor progression where epithelial cells acquire mesenchymal properties like acquiring motility which enables invasion of cells in surrounding extracellular matrix. HMGA2 led to increased TGFB signaling via suggesting an important role in EMT (Morishita et al., 2013). 

We observed an increased expression in subjects having distant metastasis and tumor recurrence on univariate analysis whereas association with local recurrence was lost upon binary logistic regression analysis. These findings suggest that HMGA2 can serve as a marker of local and distant metastatic trait of OSCC. Similar findings to our study were reported by Sakata et al who observed a strong correlation of *HMGA2* expression with distant metastasis and poor prognosis. However, they explored *HMGA2* expression via immunostaining in 110 OSCC patients (Sakata et al., 2019). 

We did not find any association of *HMGA2* expression with gender, ethnicity, smoking history, tumor size, nodal metastasis and tumor grade. Similar to us, Sakata et al also observed high expression was associated with nodal and distant metastasis but not with tumor size, stage, grade, local invasion, age or sex reemphasizing an important role of HMGA2 in tumor metastasis (Sakata et al., 2019). 

 We found that 50 % of the Gutka chewers had *HMGA2* expression signifying a poor prognostic role of gutka chewing. There is no data on effect of various chewable formulations on *HMGA2* expression but it may be assumed that gutka has some carcinogens which preferentially promote EMT and angiogenesis via turning on HMGA2.

Similar to our observation Yamazaki et al., (2013) observed a correlation with invasion and poor differentiation. They conducted research on 12 samples of head and neck cancer and surrounding normal tissue on a microarray which was confirmed on a sample of 38 tumor tissues via qRT-PCR. This study has a small sample size yet powerful results as expression was validated with two different techniques performed on same samples.

Zhao et al., (2016) also observed an up regulation of HMGA2 in their patients of tongue cancer. They investigated expression of *HMGA2* mRNA and protein in a set of 60 tongue SCCA and 20 adjacent normal tissue via RT-PCR, western blotting and immunostaining. An up regulation was associated with nodal metastasis, grade, stage and survival. They further explored the effect of HMGA2 inhibition in tongue cancer cell lines and observed decreased invasiveness. They further suggested its role as an independent diagnostic marker upon multivariate analysis. 

Morishita et al., (2013) investigated role of HMGA2 in a comprehensive research conducted in human cancer lines, mouse and human breast and colorectal tissue. They reported *HMGA2* expression near invasive fronts of tumors in both humans and mouse models. Not only did over expression of *HMGA2* imparted metastatic traits in non-metastatic breast tumor in mouse model but also suppression of HMGA2 lead to reduced tumor multiplicity.

Miyazawa et al., (2004) conducted a research on oral cancer cell lines and normal keratinocytes. Conventional PCR with specific set of primers revealed *HMGA2* expression in seven out of nine cancer cell lines while no expression was reported in normal keratinocytes. To validate the finding immunostaining of 42 OSCC tissue was done which showed negligible staining in center of tumor, higher score in invasive fronts of tumor and none in normal keratinocytes suggesting a possible role in invasion. Positive immunostaining was associated with poor survival and nodal metastasis.

Several recent studies have unveiled therapeutic potential of HMGA2 silencing in various cancers. Huang et al have suggested that ciclopirox an anti-fungal inhibited HMGA2 via directly binding to HMGA2 AT hook motifs in colorectal cancer (Huang et al., 2019). Li et al., (2016) have proposed improved treatment response upon HMGA2 inhibition in xenograft models. Moreover, transfection of HMGA2 reversed the improved clinical outcome seen with inhibition. Wang et al., (2018) discovered HMGA2 silencing inhibits ATR/Chk1 signaling pathway and represses epithelial mesenchymal transition, proliferation, migration and invasion in cervical cancer. They further observed decreased lymph node invasion and enhanced apoptosis. 

A limitation of this study was that majority of our patients were in advanced stage. Changing expression pattern with advancing tumor stage will contribute valuable information on potential use of these markers. Hence inclusion of equal number of all stages and a group with pre malignant lesion and checking expression serially would give substantial information.

It is therefore recommended to carry out serial measurements of same markers to reconnoiter their association with acquisition of tumor heterogeneity. Changing expression post treatment versus pretreatment and its impact on treatment response will edify its role in targeted therapy. Silencing *HMGA2 *expression can be a major step towards personalized medicine and monitoring response.

In conclusion, circulating HMGA2 is a predictor of local invasion and distant metastasis. It further dictates poor survival and may be used as powerful tool capable of segregating subjects with poor outcome needing more aggressive treatment. However, these findings need to be verified on a larger sample size in multicenter studies.
